# Photoacoustic Spectral Sensing Technique for Diagnosis of Biological Tissue Coagulation: In-Vitro Study

**DOI:** 10.3390/diagnostics10030133

**Published:** 2020-02-29

**Authors:** Deblina Biswas, George C. K. Chen, Hyoung Won Baac, Srivathsan Vasudevan

**Affiliations:** 1 Discipline of Electrical Engineering, Indian Institute of Technology Indore, Khandwa Road, Simrol, Madhya Pradesh 453552, India; deblina@skku.edu; 2 Department of Electrical and Computer Engineering, Sungkyunkwan University, Suwon 440-746, Korea; 3 BC Photonics Technology Co., Richmond, BC V7E 1G9, Canada; georgeckchen@live.com; 4 Discipline of Biosciences and Biomedical Engineering, Indian Institute of Technology Indore, Khandwa Road, Simrol, Madhya Pradesh 453552, India

**Keywords:** photoacoustic diagnosis, spectral response, thermal coagulation, dominant frequency

## Abstract

Thermal coagulation of abnormal tissues has evolved as a therapeutic technique for different diseases including cancer. Tissue heating beyond 55 °C causes coagulation that leads to cell death. Noninvasive diagnosis of thermally coagulated tissues is pragmatic for performing efficient therapy as well as reducing damage of surrounding healthy tissues. We propose a noninvasive, elasticity-based photoacoustic spectral sensing technique for differentiating normal and coagulated tissues. Photoacoustic diagnosis is performed for quantitative differentiation of normal and coagulated excised chicken liver and muscle tissues in vitro by characterizing a dominant frequency of photoacoustic frequency spectrum. Pronounced distinction in the spectral parameter (i.e., dominant frequency) was observed due to change in tissue elastic property. We confirmed nearly two-fold increase in dominant frequencies for the coagulated muscle and liver tissues as compared to the normal ones. A density increase caused by tissue coagulation is clearly reflected in the dominant frequency composition. Experimental results were consistent over five different sample sets, delineating the potential of proposed technique to diagnose biological tissue coagulation and thus monitor thermal coagulation therapy in clinical applications.

## 1. Introduction

In the recent years, thermal therapy has gained a lot of interest among clinicians for treating abnormal tissues such as benign and malignant tumors as it can be applied to spatially targeted parts in a minimally invasive manner [1]. This technique utilizes heating of tissues from 50 °C to 80 °C in order to obtain in-situ coagulation necrosis that leads to cell death [2]. The process of heating tissues has been performed in various techniques such as radio-frequency radiation, high-intensity focused ultrasound (HIFU), and high-power laser sources capable of forming thermal energy to the targeted region of the tissue [3,4,5]. It is essential to diagnose tissue coagulation and monitor thermally treated tissues to precisely assess a treated region and thus minimize possible damage to surrounding healthy tissues. In this regard, several modalities have been explored, such as magnetic resonance imaging (MRI), ultrasound imaging (US), and optical techniques such as optical coherence tomography (OCT) and optical spectroscopy [6,7,8,9,10]. Although these techniques have their own merits for monitoring thermal therapy, they have certain limitations. For example, MRI provides high resolution and contrast but suffers from high cost and acquisition time. In contrast, ultrasound allows real-time diagnosis in low cost, but its relatively poor imaging contrast often makes diagnostic decision obscure for thermally coagulated tissues. Subsequently, optical techniques such as OCT and optical coherence elastography (OCE) have been extensively utilized for tissue characterization based on mechanical properties with micro-scale spatial resolution [9,11]. Since tissue coagulation significantly increases optical scattering, the penetration depth in these optical techniques has been limited only to a few mm [12]. Hence, it would be highly beneficial if noninvasive differentiation between normal and coagulated tissues could be performed with high contrast and low cost to monitor the therapeutic efficiency of thermally treated tissues and then diagnose the coagulated tissues.

Here, we propose a photoacoustic (PA) spectral sensing technique for in-vitro quantitative differentiation of intact and coagulated chicken tissues based on tissue elasticity. As a noninvasive and nonionizing diagnosis technique, this utilizes nano-second laser pulses to generate bipolar acoustic waves thermo-elastically from light-absorbing tissues, which are referred to as PA response [13]. Intrinsic properties of the target tissue, such as optical absorption and tissue elasticity, can be characterized by PA responses in terms of peak pressure amplitude, pulse width, and relaxation time [14,15,16]. Since thermal coagulation of tissues induces significant deformation in tissue elasticity, intact and coagulated tissues can be differentiated sensitively by using their PA responses. Here, we measure time-domain PA waveforms using intact and coagulated excised chicken tissues and then characterize their frequency spectra. A dominant frequency component of PA frequency spectrum is introduced to quantitatively characterize coagulation-induced tissue hardness. A significant high-frequency shift of more than 1 MHz is observed for differentiation. Two types of tissues with different initial elasticity (excised chicken liver and muscle) are used to prove the validity of the PA spectral sensing technique. The consistency of the results is also verified by applying the technique to five sets of animal tissues.

## 2. Materials and Methods

### 2.1. Sample Preparation for Coagulation Study

**Figure 1:**
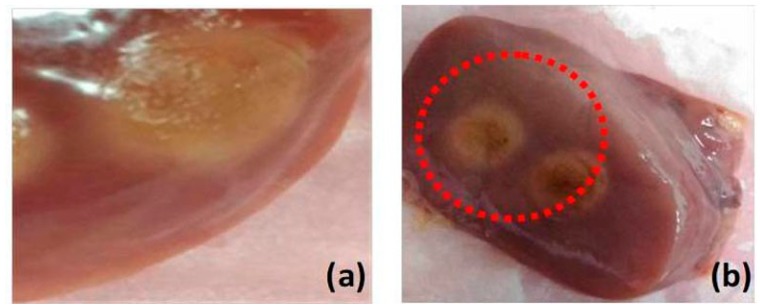
Photograph of chicken tissue (**a**) muscle and (**b**) liver. Discolored part indicates the coagulated regions.

For this study, we prepared tissue samples from five chicken livers and five chicken muscles. The samples are normal chicken meat available in market. For thermal therapy of the tissues, these samples were exposed to a nanosecond pulsed laser beam (either 532- or 1064-nm wavelength available) with an optical fluence of 100 mJ/cm^2^ for 1 min. In order to ensure coagulation of tissue samples, we used the laser beam with 532-nm wavelength to irradiate the liver tissues and 1064-nm wavelength to irradiate the muscle tissues. Both tissues were exposed to the identical optical fluence. The samples under laser irradiation were carefully monitored to ensure coagulation and avoid carbonization or evaporation. Figure 1 shows examples of coagulated liver and muscle tissues. Then, the samples were coated with a thin layer of ultrasound gel and wrapped with a parafilm avoiding bubble formation. Thereafter, the samples were placed to the bottom of water tank.

### 2.2. Experimental Setup

**Figure 2:**
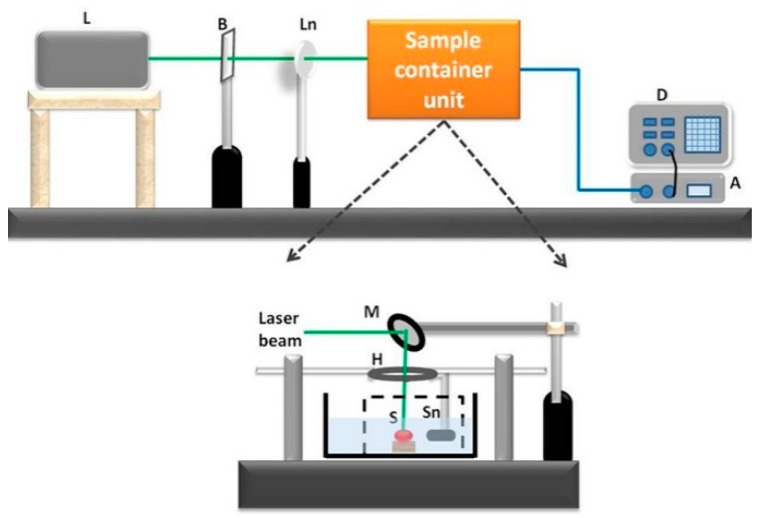
Schematic of PA spectral sensing experimental setup (L-laser; B-beam splitter; Ln-lens; D-digitizer; M-mirror; H-holder; S-sample; Sn-sensor).

The experimental schematic is shown in Figure 2. Tissue samples were irradiated with an Nd:YAG pulsed laser beam (Ekspla NT342c; 5-ns width, 10-Hz repetition rate, 2 mJ/cm^2^ optical fluence). Under laser irradiation with the short temporal pulses, the tissues absorbed the optical energy and generated thermoelastic volume expansion before significant heat diffusion occurred. These signals were acquired by an ultrasound sensor (Panametric V383; centre frequency 3.5 MHz; unfocused), which was placed approximately 3 cm away from the tissue sample. The sensor output was connected to a digitizer (NI PXI5124), which acquires the time-domain signal at 200 Msps sampling rate. A time average was performed by using 200 waveforms in order to improve the signal-to-noise ratio.

### 2.3. Frequency Spectral Analysis of PA Response

For extraction of frequency spectral parameters from obtained PA response, Fast Fourier Transform (FFT) was applied. Prior to performing FFT, the background noise was subtracted from the signal to obtain the true PA response of the sample. Subsequently, PA responses were normalized to eliminate effect of optical absorption and other experimental parameters. A dominant frequency of the PA frequency spectrum was utilized as a key parameter for elasticity to differentiate normal and coagulated chicken tissues.

## 3. Results and Discussion

**Figure 3:**
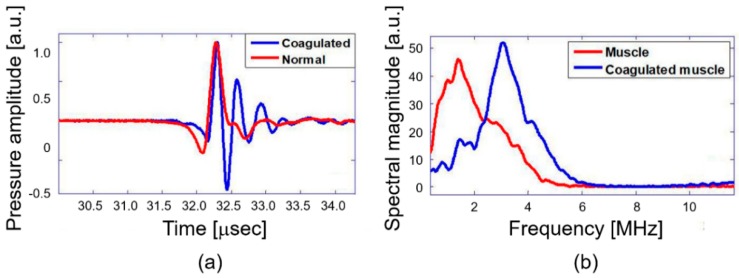
Chicken muscle tissue normal and coagulated: (**a**) photoacoustic (PA) response and (**b**) PA frequency spectrum.

**Figure 4:**
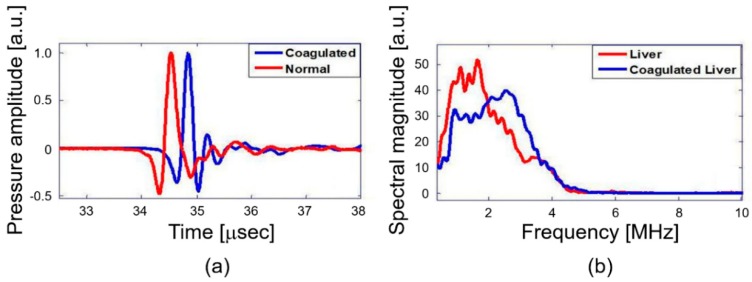
Chicken liver tissue normal and coagulated: (**a**) PA response and (**b**) PA frequency spectrum.

The primary objective of this study is quantitative differentiation of normal and coagulated tissue based on elastic property of the samples. Hence, the PA spectral sensing technique was applied onto normal and coagulated chicken liver and muscle tissue to obtain PA frequency spectral parameter (dominant frequency). This feature served as the fingerprint for normal and coagulated tissues. For this purpose, PA responses were acquired from three different regions of normal and coagulated part of each sample by applying the developed setup as described in Figure 2. Figure 3a illustrates PA responses acquired from normal and coagulated chicken muscle tissues that show a distinct change in PA responses. The normal tissue exhibits significant change in relaxation time and less oscillation compared to coagulated muscles. For example, only a single oscillation before attaining stability is observed in the normal tissue, whereas the coagulated tissue delineates three significant oscillation peaks. The change in the PA response is clearly reflected in the frequency spectrum as shown in Figure 3b. The coagulated muscle elucidates the dominant frequency of 3.3 MHz, whereas the normal muscle exhibits 1.7 MHz. A significant two-fold enhancement in the dominant frequency is observed between normal and coagulated muscle. We also characterized normal and coagulated chicken liver tissues with the PA spectral sensing technique. This verifies the applicability of our technique into different tissue types. Figure 4a illustrates PA responses obtained from the normal and coagulated liver tissues. A significant disparity is observed in relaxation time and oscillation between two samples. The PA frequency spectrum in Figure 4b also illustrates a 1-MHz increase in the dominant frequency for coagulated liver compared to the normal case.

**Figure 5:**
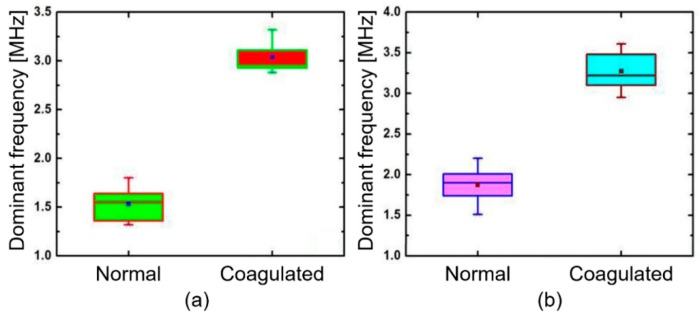
Comparison of dominant frequencies obtained from four muscle and liver tissues: (**a**) muscle and (**b**) liver. The shaded box represents a 25–75% range of each data set. The line and the dot within each box show the median and the mean value, respectively.

To confirm the reproducibility, the PA spectral sensing experiments were repeated with four other muscle and liver tissues, as shown in Figure 5a,b. This exhibits the dominant frequency of normal muscle tissues that lies between 1.3 and 1.7 MHz, whereas coagulated tissues exhibits the range between 2.8 and 3.3 MHz. Additionally, the dominant frequency of normal liver lies between 1.5 and 2.2 MHz whereas coagulated liver tissues have a range between 2.9 and 3.6 MHz.

Such repeated results suggest that the PA spectral parameter (i.e., dominant frequency) can be correlated with elastic properties of normal and coagulated tissues. A PA technique refers to an acoustic wave generation process due to thermo-elastic volume expansion by optical absorption with pulsed laser beams. The process involves a localized temperature increase when the laser pulse duration is shorter than thermal and stress confinement time [17]. A transient pressure rise occurs due to the temperature enhancement, and thus the sample undergoes thermo-elastic expansion. In this sense, the thermal expansion of the sample is directly associated with the initial pressure rise. It is this initial pressure source that can be treated as a strain source. The variation in the elastic modulus of irradiated tissue dictates the strength of the strain source generated due to light absorption. Due to the pressure variation, acoustic waves (i.e., PA response) with wide frequency ranges are generated at the illuminated spot [18,19]. Subsequently, theses acoustic waves traverse through the surrounding tissue and the guided medium. The sound speed of these acoustic waves (*V_s_*) is directly proportional to its angular frequency (*ω*) for constant wavenumber (*k*_0_), which can be represented as follows [20]:

(1) $$k_{0}=\frac{\omega }{V_{s}}$$

where *ω* = 2π*f* and *f* is the linear frequency. As the sound speed can be increased with the bulk modulus (*K*) through *K* = *V_s_*^2^*ρ* [17,21,22], a frequency shift to the higher range can be obtained in the PA response for the constant wavenumber. For thermally coagulated tissues, this effect was experimentally confirmed as an enhancement in the dominant frequency component in the PA frequency spectrum.

Since tissues undergo drastic changes in cellular conformation during thermal coagulation, the mechanobiological property also alters significantly [2,23,24]. For laser-induced thermal coagulation of tissues, we used the optical fluence of 100 mJ/cm^2^ for 1 min (i.e., 600 laser pulses). This condition exceeds an optically deposited energy required for full coagulation for both muscle and liver tissues. This leads to the denaturization process of collagen, which can be visually confirmed as discoloration of coagulated tissue area [25]. During denaturization, the fibril fibers shrink to maintain the organization of micro-fibril [26,27]. The shrinkage of fibril fibers can induce oscillation in the PA response of coagulated tissues, causing the shift of dominant frequency, as confirmed in Figure 3 and Figure 4. The shrinkage also suggests a significant increase in the density of coagulated tissues, compared to normal tissues, which eventually enhances the elastic modulus of coagulated tissues [28,29]. Interestingly, Fatemi et al. reported a six-fold increase in shear stiffness of bovine muscle tissues during irreversible protein denaturization [29]. Similarly, another study indicated that coagulated pig liver tissues exhibit an elasticity value of 38.5 ± 2.5 kPa whereas normal liver tissues exhibit only 6.4 ± 0.3 kPa [28]. These studies promptly demonstrate a significant contrast between normal and coagulated tissue stiffness.

Utilizing such strong contrast in normal and coagulated tissue elasticity, conventional ultrasound elastography and MR-guided elastography techniques have been explored to differentiate normal and coagulated tissues [28,29]. Despite promising results, the US elastography requires an external force application to measure the variation in tissue stiffness, which puts limitations on ex-vivo and/or specific in-vivo applications [30]. The MR-based approach suffers from its classical issue of high cost and limited availability [31]. Additionally, OCT has been used to measure micro-scale displacement in retinal tissue [9]. The elastic property of tissue has been characterized by OCE with several microns of spatial resolution, allowing highly localized examination [10]. However, these optical techniques suffer from low penetration depth of a few mm, which hinders their applications into deep tissue or organs [12]. Previously, PA techniques have been also investigated to study thermal coagulation of tissues based on changes in PA signal amplitudes due to changes in optical properties of tissues during coagulation [32]. However, such PA amplitude-based differentiation is often not reliable because the PA signal strength depends nonlinearly on an input laser energy and a distance between tissue sample and acoustic sensor [15]. In this regard, our proposed PA spectral sensing technique is more robust and predictable as it utilizes the frequency of PA response that is relatively less sensitive to experimental parameters, compared to the case of PA pressure amplitudes. Moreover, the proposed technique relies on changes in the elastic property of tissue that serves as a potential fingerprint in coagulated tissue study. Our approach has been previously adopted to diagnose different pathological conditions involving minute changes in elastic properties [15,33]. This exhibits the versatility and sensitivity of our PA spectral sensing technique. The present study can be further improved by using broadband and high-frequency transducers together with an advanced signal processing technique to perform in-vivo (high penetration depth of several cm) and real-time studies that would pave the way for clinical applications.

## 4. Conclusions

The PA spectral sensing technique was proposed to differentiate normal and coagulated excised chicken tissues depending upon the elastic property of tissues. A clear disparity in the dominant frequency of PA frequency spectrum was observed between normal and coagulated chicken liver tissues and also between normal and coagulated muscle tissues. The coagulated tissues illustrated approximately two-fold enhancement in the dominant frequency compared to the normal tissues. This was explained by the enhanced bulk modulus, which increased the sound speed and then the dominant frequency. The obtained results were consistent for five different tissues collected from five different animals. We expect that our technique can be applied to monitor thermal therapy for noninvasive and real-time applications.
